# Effects of L-arginine pre-treatment in 1-methyl-4-phenyl-1,2,3,6-tetrahydropyridine-induced Parkinson’s diseases in Balb/c mice

**Published:** 2015-10-07

**Authors:** Javad Hami, Mehran Hosseini, Sekineh Shahi, Nassim Lotfi, Abolfazl Talebi, Mohammad Afshar

**Affiliations:** 1 Department of Anatomy, School of Medicine, Birjand University of Medical Sciences, Birjand, Iran; 2 Research Centre of Experimental Medicine AND Department of Public Health, Deputy of Research and Technology, Birjand University of Medical Sciences, Birjand, Iran; 3 Department of Biology, School of Sciences, Payame Noor University, Tehran, Iran; 4 Department of Anatomy, School of Medicine, Birjand University of Medical Sciences, Birjand AND Department of Anatomy and Cell Biology, School of Medicine, Mashhad University of Medical Sciences, Mashhad, Iran; 5 School of Medicine, Birjand University of Medical Sciences, Birjand, Iran

**Keywords:** Parkinson Disease, 1-Methyl-4-phenyl-1, 2, 3, 6-tetrahydropyridine, BALB C Mice, Protective Agents

## Abstract

**Background:** Parkinson’s disease (PD) is a common neurodegenerative disease resulting from the degeneration of dopaminergic (DA) neurons in the substantia nigra pars compacta (SNc). Increasing evidence demonstrated that mice treated intranasally with 1-methyl-4-phenyl-1,2,3,6-tetrahydropyridine (MPTP) suffered impairments in motor functions associated with disruption of DA neurons in SNc conceivably analogous to those observed in PD. L-arginine has been proposed as a novel neuroprotective agent that plays protective roles in several models of neuronal cellular damage. This study aimed to evaluate the effects of L-arginine on the numerical density of dark neurons (DNs) in the SNc of Balb/c mice subjected to MPTP administration.

**Methods: **In the present study, we demonstrated that repeated treatment with L-arginine (300 mg/kg, i.p.) during 7 consecutive days attenuated the production of DNs in SNc of adult male Balb/c mice infused with a single intranasal administration of MPTP (1 mg/nostril).

**Results: **Pre-treatment with L-arginine significantly decreased the numerical density of DNs in SNc of mice 21 days after intranasal MPTP administration.

**Conclusion:** This investigation provides new insights in experimental models of PD, indicating that L-arginine represents a potential neuroprotective agent for the prevention of DA neuron degeneration in SNc observed in PD patients.

## Introduction

Parkinson’s disease (PD) is the second most common neurodegenerative and age-related disease that usually affects people over the age of 50.   1^,^^2^ PD is a slowly progressing disorder resulting in degeneration of dopaminergic (DA) neurons in the substantia nigra pars compacta (SNc), a region of the brain that controls movement. The neuronal death in SNc leads to impaired motor functions such as tremor, bradykinesia, akinesia, rigidity, and postural instability.^   3 ^ While great advances are being made in our understanding of the risk factors underlying PD that may soon allow for the clinical use of preventive pharmaceuticals, at this time these do not exist.^   4 ^ Even though etiology of disease remains unknown, there are well-known mechanisms involved in the pathogenesis of DA nigrostriatal degeneration of PD patients, including apoptosis, oxidative stress, and mitochondrial dysfunction.^3^^,^^5^ These interrelated events finally lead to neuronal death by apoptosis; hence, anti-apoptosis strategies could, in principle, prevent, or delay the progression of PD.^        6 ^

Recent experimental and epidemiological studies suggest that intranasal (i.n.) infusion of several environmental agents, including viruses^   7 ^ or cadmium,^   8 ^ or inhalation of aluminum^   9 ^ or manganese, may contribute to PD pathogenesis.^        10 ^ Sometimes such agents may enter the brain via the olfactory neuroepithelium, a concept termed the olfactory vector hypothesis.^        11 ^ In accordance with this hypothesis, several studies have shown that approximately 90% of patients with early-stage PD exhibit olfactory dysfunction^11^^,^^12^ and that the olfactory bulb is among the first brain structures to exhibit PD-related pathology, occurring pre-clinically before the classic disease motor signs.^   13 ^

In a series of earlier human and experimental studies, administration of 1-methyl-4-phenyl-1,2,3,6-tetrahydropyridine (MPTP), a systemic neurotoxin, causes a specific loss of DA neurons in the nigrostriatal system, that recapitulated the DA neuron degeneration seen in idiopathic PD patients.^        14 ^ Therefore, in the recent works, the MPTP mouse model has become the most commonly used animal model of PD.^6^^,^^15^^-^^20^ In addition, the some of them demonstrated that low concentrations of MPTP can enter the brain via the olfactory mucosa and alter DA function in a range of brain structures.^16^^-^^20^ Because of safety considerations, the i.n. administration of MPTP, which is not constrained by such factors, may be more effective in getting higher levels of MPTP into the brain and to induce alterations in central nervous system structure and function.^16^^-^^20^ 

When MPTP is injected into animal, the chemical penetrates the brain through the blood-brain barrier and is metabolized to 1-methyl-4-phenyl1-2,3-dihydropyridium (MPP+) by monoamine oxidase-B enzyme in glia. MPP+ has high affinity for the dopamine transporter (DAT) on DA cells, and is taken up into the cell.^   21 ^ MPP+ is then released from the glia and enters neurons via the DAT on DA cells.^        22 ^ MPP+ is then accumulated in the mitochondria and creates further neuronal damage through the activation of reactive microglia and subsequent generation of free radicals.^5^^,^^23^ By seven days post-administration of MPTP, a significant loss of DA neurons in the SNc is evident, along with a significant reduction of DA production in the terminal field within the striatum.^        24 ^ Thus, MPTP administration to animals induces a DA neuron loss that mirrors the loss seen in end-stage PD.

L-arginine is a semi-essential amino acid and has different roles in the normal brain functioning. L-arginine is oxidized to nitric oxide (NO) in a NADPH-dependent reaction by the action of the enzyme nitric oxide synthase (NOS). L-arginine and NO play a modulatory role in the brain, and are involved in synaptogenesis, synaptic plasticity, neurogenesis, neuroprotection, memory and learning function, and neuroendocrine secretion.^25^^,^^26^ It also has been shown that NO is synthesized by neurons as a response to the activation of N-methyl-D-aspartate (NMDA) receptors by the excitatory amino acid glutamate,^27^^-^^29^ and leads to the formation of guanosine 3',5'-cyclic monophosphate (cGMP) in the brain.^28^^,^^30^ Further, other studies have demonstrated the feedback inhibition of NMDA receptors by NO.^31^^,^^32^ Experimental evidence has demonstrated that NO is involved in NMDA receptor-mediated neurotoxicity^        33 ^ and in the neuronal death that occurs after focal cerebral ischemia.^34^^,^^35^ 

L-arginine and NO can also influence the immune system by playing a key role in regulating inflammatory processes and redox stress.^36^^,^^37^ It also promotes easy and efficient flow of blood through the blood vessels going to the brain.^38^^,^^39^ L-arginine is also implicated in the pathophysiology of some neurodegenerative disease (i.e., Alzheimer’s disease), although it's precise role remains to be determined.^40^^,^^41^ Moreover, there are no reliable proofs yet with the use of L-arginine to prevent or treat PD disease. Although, it had shown some ability in improving certain conditions.^        42 ^

In previous experimental studies, dark neurons (DNs) productions have been reported in the brain of animals exposed to various pathological conditions.^43^^,^^44^ DNs are the final product of a series of physico-chemical reactions initiated from extracellular milieu and propagate into the neuron.   ^45^^-^^49^ Morphologically DNs are characterized by at least six features namely: hyperbasophilia, argyrophilia, disappearance of antigenicity, ultrastructural compaction, volume reduction and increased electron density.^        43 ^ In addition, the morphological study of DNs by transmission electron microscopy showed chromatin changes, darkness, and shrinkage and swelled mitochondria.^   46 ^ It is believed that these types of neurons are in recovering phase (reversible type) in contrast to real DN (dead or irreversible).^45^^,^^47^^-^^49^ These kind of degenerating neurons have been reported in Huntington disease, epilepsy, spreading depression, and also in aging process.   ^44^^,^^50^ 

Since L-arginine and its product, NO, exert such a range of critical roles in regulating physiological functions of the brain, we hypothesize that L-arginine can possibly prevent the MPTP-induced neurodegeneration in the SNc of mice. So, this study was designed to evaluate the effects of L-arginine on the numerical density of DNs in the SNc of Balb/c mice subjected to MPTP administration.

## Materials and Methods

Healthy adult male Balb/c mice (20-30 g body weight, 6-8 weeks old) were purchased from the Experimental Animal Facility of Birjand University of Medical Sciences, Iran. The animals were housed in polypropylene cages (four per cage) under controlled temperature and light conditions (22 ± 3 °C, 40-70% relative humidity, 12 hours light phase with daylight). They were fed with standard pellet diet (Javaneh co., Iran) and water ad libitum. All procedures involving animals were conducted in accordance with the Guide for the Care and Use of Laboratory Animals of the Birjand University of Medical Sciences. All efforts were made to minimize animal suffering and to reduce the number of animals used.

Mice were randomly assigned to four equal groups (n = 7 each):

1. Model control (MPTP) group: mice were administrated intranasally with a single dose of MPTP (Sigma-aldrich, St. Louis, MO, USA; dissolved in saline 0.9%) at the dose of 1 mg/nostril.^18^^,^^51^ 
2. Sham control group: mice were administrated intranasally with a same dose of vehicle (saline 0.9%).
3. L-arginine - treated model (L-arginine treated PTP) Group: mice received intraperitoneally L-arginine (Sigma-aldrich, St. Louis, MO, USA; 300 mg/kg dissolved in saline 0.9%) once daily for 1 week starting from 3 days before MPTP administration.
4. L-arginine control group: mice only received intraperitoneally L-arginine (Sigma-aldrich, St. Louis, MO, USA; 300 mg/kg dissolved in saline 0.9%) once daily for 1 week.

**Figure 1:**
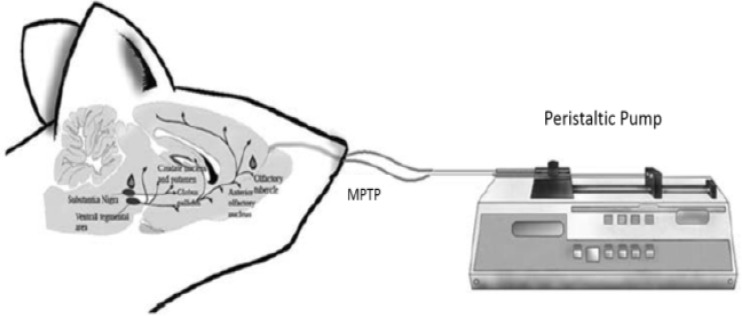
A schematic procedure of the intranasal administration of 1-methyl-4-phenyl-1,2,3,6-tetrahydropyridine in mice

MPTP (1 mg\nostril) was administered by i.n. route according to the procedure previously described^16^^-^^18^ and modified in our laboratory. Briefly, mice were lightly anesthetized with xylazine/ketamine (10-75 mg/kg body weight, intraperitoneal injection) and a 7 mm piece of PE-10 tubing was inserted through the nostrils. The tubing was connected to a calibrated peristaltic pump set at a flow rate of 12.5 IU/minutes (Figure 1). The MPTP was dissolved in saline at a concentration of 20 mg/ml, after which it was infused in 1 minute intervals for 4 minutes (6 seconds pump on and 54 seconds pump off).

The control solution consisted of saline. Animals were given a 1 minute interval to regain normal respiratory function and then this procedure was repeated with infusions administered through the contralateral nostrils.

Twenty-one days after the MPTP administration, mice were anesthetized with chloral hydrate (100 mg/kg). The mice were subjected to thoracotomy and perfusion with ice-cold 0.9% sodium chloride 50 ml, then with 4% paraformaldehyde 100 ml in 0.01 M phosphate buffered saline (PBS) through the left ventricle. After fixation, the brains were removed immediately and post-fixed overnight at room temperature in the following fixative: 10% formaldehyde in 0.01 M PBS.

Following fixation, samples were dehydrated using an ascending ethanol series, cleared in xylene and infiltrated with paraffin. They were then embedded in paraffin and sectioned through the SN coronally at 5 μm thickness using rotary microtome (Leica, Germany). All of the sections containing SN^   52 ^ were mounted on slides. Sections were stained with 1% toluidine blue in 1% sodium borate for 1 minute at 60 °C.

DNs in SNc were counted by an investigator blinded to the protocol treatment, using the optical dissector technique described in detail by Gundersen et al.^   53 ^ The optical dissector technique eliminates bias in counting as a result of cell size and shape. Briefly, DNs were counted as they came into focus while scanning through the section.

**Figure 2:**
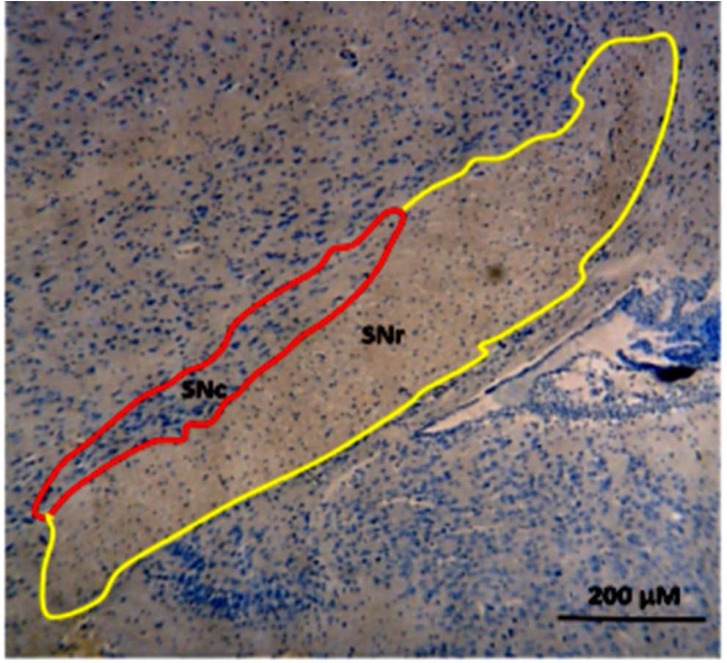
Photomicrograph of coronal section from the mice substantia nigra (SN) sub-regions [SNc: Substantia Nigra pars compacta (Red); SNr: Substantia Nigra pars reticulata (Yellow)] illustrating 1-methyl-4-phenyl-1,2,3,6-tetrahydropyridine-induced dark neurons production in SNc sub-region where used for the stereological study; Regional boundaries were determined by cross-referencing with the atlases of Paxinos and Watson

For each section, 4-6 unbiased counting frames were sampled in a systematically random fashion inside the area of SNc. The preparations were examined under a light microscope using a ×60 objective lens (UPlanFI, Japan) and images were transferred to a computer using a high-resolution camera (BX51, Japan). The number of DNs was counted using a 10,000 μm^2^ counting frame. The mean numbers of neurons per unit area (NA) in SNc were calculated using the formula as follows:

$N_{A}=\frac{\sum \overset{\text{̅}}{Q}}{a|\mathrm{f.}\sum P}$

In this formula “$\sum \overset{\text{̅}}{Q}$”is the summation of counted DNs appeared in sections, “a/f” is the area associated with each frame (10,000 μm^2^), “∑P” is the sum of frames associated points hitting the reference space.

The numbers of 8-10 sections from each animal were averaged, and the data from 7 animals of each group were presented as means ± standard deviation. Results were analyzed using one-way ANOVA, followed by Tukey’s post-hoc test for multiple comparisons between different groups studied. The level of statistical significance was set at P < 0.05. SPSS software for Windows (version 19, SPSS Inc., Chicago, IL, USA) was used to perform the total statistical analysis.

## Results

To explore the neuroprotective effects of L-arginine against MPTP-induced neuronal loss, Toluidine Blue staining was used to examine the numerical density of Dark degeneration neurons in the SNc of Balb/c mice. Normal cells showed round and pale stained nuclei with a distinct nucleolus. The shrunken cells after MPTP administration with the morphological features of pro-apoptosis such as nuclear shrinkage and condensed chromatin were counted as DNs. To determine the numerical density of DNs in the SNc of Balb/c mice, we traced the boundaries for SNc as in figure 2. The numerical density of DNs were stereologically counted in SNc of mice in different studied groups.

**Figure 3:**
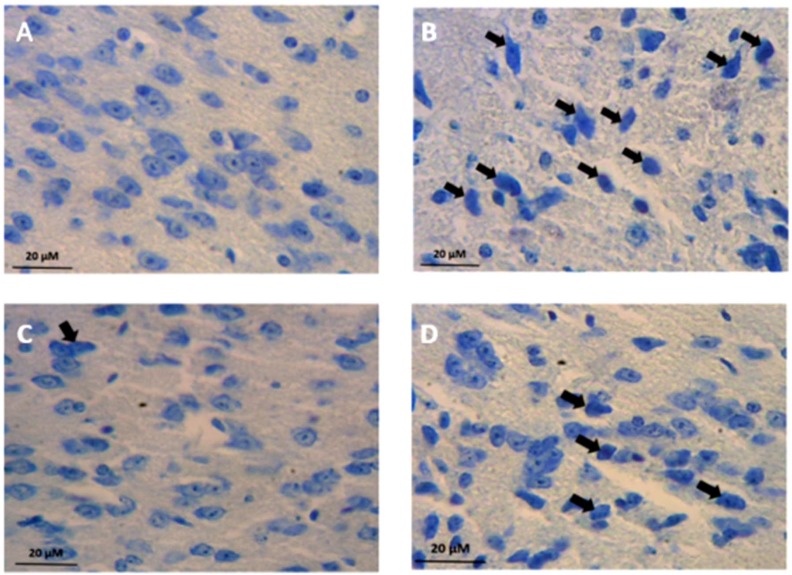
Photomicrographs showing distribution of dark neurons (DNs) in Substintia Nigra pars compacta (SNc) subdivisions of Balb/c mice in the Sham-control (a), 1-methyl-4-phenyl-1,2,3,6-tetrahydropyridine (MPTP) (b), L-Arginie-control (c), and L-arginine plus MPTP (d) groups. DNs pointed with black arrows. As shown the distribution of DNs in SNc sub-region were strikingly increased in MPTP and L-arginine plus MPTP group animals, compared to Sham-control and L-arginine-control Balb/c Mice

**Figure 4:**
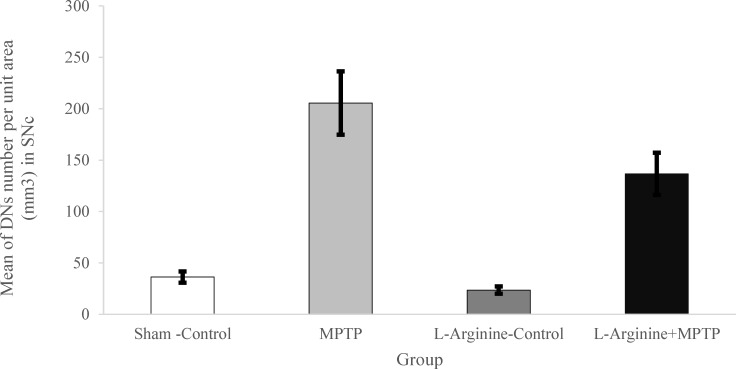
Mean of dark neuron (DN) numbers per unit area in the Substintia Nigra pars compacta (SNc) subdivisions of Balb/c mice and its comparison in the different studied groups. The data show that the mean number of DNs per unit area in 1-methyl-4-phenyl-1,2,3,6-tetrahydropyridine (MPTP) group significantly increased in SNc comparing to L-arginine-Control and Sham-Control Balb/c Mice. Evaluation of neuroprotective effects on DN production in SNc sub-region revealed a significant reduction in the mean number of DNs in L-arginine plus MPTP groups

^*^ P < 0.05 significant difference

In sham-control and L-arginine-control groups, there were a few numbers of DNs in SNc of the Balb/c mice (Figures 3 a, c, and 4). MPTP administration induced severe DNs production. Our results revealed a marked increase in the number of DNs in SNc of the Balb/c mice in MPTP group when compared with both sham-control and L-arginine-control groups (P < 0.05 and P < 0.01, respectively) (Figures 3 a, b, d, and 4). In addition, the number of DNs in the L-arginine plus MPTP group also increased significantly when compared with both control–sham and control groups (P < 0.05 and P < 0.01, respectively) (Figures 3 a, c-d, and 4).

Nevertheless administration of L-arginine (300 mg/kg; i.p.) once daily for 7 days starting from three days before MPTP administration significantly decreased the numerical density of dark degenerating neurons in SNc sub-region of SN of the Balb/c mice (P < 0.05) (Figures 3a, d, and 4). We found a statistical decrease in the number of DNs in the SNc in the L-arginine plus MPTP group Balb/c mice comparing to the MPTP group (P < 0.05) (Figures 3a, d, and 4).

## Discussion

PD is one of the most common neurological disorders that is mainly characterized by problems with body movements.^1^^-^^3^ Regarding PD symptoms, an increasing number of studies have demonstrated that PD seems to be a multidimensional disease, and besides motor deficits, it is associated with a number of sensorial, cognitive and emotional disturbances.^        54 ^ In this context, a recent series of studies demonstrated that a single i.n. infusion of MPTP in rodents produces diverse signs of PD such as impairments in motor, cognitive, emotional, and olfactory functions.^16^^,^^18^^,^^19^ 

Preventive or therapeutic strategies that stop or even slow the progress of neurodegenerative disorders such as PD are expected to have a major impact on the prevention or treatment of these diseases.^   55 ^ The results of this study show novel neuroprotective effects of L-arginine against MPTP-induced neurodegeneration in SN of Balb/c mice. To our knowledge, this is the first report investigating the neuroprotective effects of L-arginine in animal model of PD.

The current hypothesis about the mechanisms by which neurons come into apoptotic or necrotic process of degeneration has led to the belief that the use of drugs modulating the function of glutamate NMDA receptors may have beneficial effects in PD cases.^        56 ^ In this context, there is increasing evidence of the neuroprotective effects of L-arginine, which among other possible targets blockades NMDA receptors such as Mg^2+^ ions, against different insults of the CNS.^57^^-^^59^ Recent studies on laboratory animals revealed that the administration of L-arginine has potential therapeutic importance, including anticonvulsant, anxiolytic and antidepressant-like actions.^57^^-^^59^ 

L-arginine is a normal constituent of the body and is found in both enteral and parental nutrition formulas, little experience is available about the pharmacology of L-arginine administration in the doses given in experimental studies, especially in neurodegenerative diseases.^42^^,^^60^ Nevertheless, there is sufficient evidence suggesting that the 300 mg/kg dose of L-arginine in the rodents provides the best results.^        60 ^

In this study, the repeated treatment with L-arginine (300 mg/kg, i.p.) during 7 consecutive days was able to decrease significantly the numerical density of DNs in SNc of Balb/c mice administrated intranasally with MPTP (1 mg/nostril). These data corroborate the neuroprotective potential of L-arginine (300 mg/kg, i.p.) in PD, since it attenuated the DA cell loss in the SNc of Balb/c mice infused intranasally with MPTP (1 mg/nostril).

In earlier investigations, administration of L-arginine has been shown to increase cerebral blood flow (CBF) and reduce neurological damage after experimental traumatic brain injury (TBI).^60^^-^^63^ A study by Cherian et al., the researchers found that the L-arginine administration (300 mg/kg, i.v., 5 minutes after the brain injury) restores CBF to near pre-injury levels and significantly reduces the volume of contused brain.^        63 ^ In experimental TBI models and in some cerebral ischemia models also similar neuroprotective effects have been observed with administration of L-arginine.^60^^,^^62^^,^^63^ As a result of these observations, L-arginine has become an interesting potential therapeutic agent for improving cerebral perfusion after TBI.

The neuroprotective effects of L-arginine may result from different mechanisms including blocking of NMDA receptors,^57^^-^^59^ inhibition of NOS,^        64 ^ oxygen radical scavenging^        65 ^ and protection against mitochondrial membrane potential collapse.^   66 ^ However, the sequence of events leading to the protective effects of L-arginine against cell damage has not been fully elucidated. Previous studies have demonstrated that MPTP decreases glutamate uptake by astrocytes in cell culture.^   67 ^ Therefore, one possible mechanism by which L-arginine may exert protective effects against MPTP neurotoxicity may be due to the modulation of glutamate reuptake into neural cells, the main mechanism responsible for decreasing extracellular glutamate levels, thus attenuating glutamate neurotoxicity.

In addition, the neuroprotective effects of L-arginine administration could occur from its effects on the vasculature,^   68 ^ including that L-arginine is essential for the function of certain KATP channels.^   69 ^ Some of neuroprotective effects L-arginine are also presumed to occur via production of NO, as L-arginine is the precursor of NO in the reaction mediated by the enzyme NOS. NO is produced by many different tissues and has numerous physiological and pathological effects.^39^^,^^42^^,^^62^ In the brain, NO plays a role as a neurotransmitter by stimulating soluble guanylyl cyclase to form the second messenger molecule, cGMP in the target cells.^   26 ^ Experimental studies have well documented the synthesis of NO in the brain, and its role in a variety of neuronal functions including learning and memory processes, cortical arousal, and blood vessel dilatation and immune response.^   26 ^

NO is also a potent vasodilator and inhibits the platelet aggregation and leukocyte adhesion and may improve blood flow by preventing microvascular plugging by platelets and leukocytes.^   70 ^ NO inhibits Ca^2+^ influx through the NMDA receptor and may limit glutamate neurotoxicity in cerebral ischemia.^70^^,^^71^ 

On the other hand, agmatine, formed by the decarboxylation of L-arginine by arginine decarboxylase, has been shown to be neuroprotective in experimental brain trauma and ischemia models.^        51 ^

Recently, agmatine has been proposed as a novel neuromodulator that plays protective roles in several models of neuronal cellular damage.^51^^,^^72^ A study by Matheus et al.^        51 ^ demonstrated that treatment with agmatine (30 mg/kg, i.p.) during 5 consecutive days increased the survival rate of old C57BL/6 female mice infused with a single i.n. administration of MPTP (1 mg/nostril), improving the general neurological status of the surviving animals. Moreover, pretreatment with agmatine was found to attenuate memory and locomotor activity, impairments observed at different periods after i.n. MPTP administration. They also reported that behavioral benefits of agmatine were accompanied by a protection against the MPTP induced loss of DA neurons in the SNc of aging mice. The researchers claimed that agmatine represents a novel potential therapeutic tool for the management of cognitive and motor symptoms of PD, together with its neuroprotective effects.

Of high importance, the administration of L-arginine demonstrated its neuroprotective properties as previously described in several models of neuronal damage.^63^^,^^73^ These results corroborate recent findings on L-arginine neuroprotection in cellular models of neurodegenerative diseases.^        42 ^ Taken together, these results suggest that L-arginine may represent a potential disease-modifying therapy for PD.

## Conclusion

The present findings reinforce i.n. MPTP administration as a valuable rodent model for testing novel palliative and neuroprotective compounds for PD. More importantly, the present study provides the first preclinical data indicating that repeated systemic treatment with L-arginine prevents DA cell loss in the SNc of mice submitted to an experimental model of PD. These results provide new insights in experimental models of PD, indicating that L-arginine may represent a new neuroprotective agent for the prevention of DA neuron degeneration observed in PD patients.

## Notes:

**How to cite this article: **Hami J, Hosseini M, Shahi S, Lotfi N, Talebi A, Afshar M. Effects of L-arginine pre-treatment in 1-methyl-4-phenyl-1,2,3,6-tetrahydropyridine-induced Parkinson’s diseases in Balb/c mice. Iran J Neurol 2015; 14(4): 195-203.
